# Influences of oxygen and temperature interaction on the antibacterial activity, antioxidant activity, serum biochemical indices, blood indices and growth performance of crucian carp

**DOI:** 10.7717/peerj.14530

**Published:** 2023-01-03

**Authors:** Bin Wang, Hanping Mao, Jian Zhao, Yong Liu, Yafei Wang, Xiaoxue Du

**Affiliations:** 1 School of Agricultural Engineering, Jiangsu University, Jiangsu, Zhenjiang, China; 2 College of Biosystems Engineering and Food Science, Zhejiang University, Zhejiang, Hangzhou, China

**Keywords:** Environmental factors, Growth performance, Physiology, Biochemistry

## Abstract

The well-being of fish used in aquaculture is of great interest. Oxygen and temperature are the main factors affecting the welfare of the crucian carp (carassius); however, there are few studies on the combined effects of these on the species. Therefore, this study investigated the impact of different temperatures (18 °C, 24 °C, 30 °C) and oxygen concentrations (2.1 mgL^−1^, 5.4 mgL^−1^, 9.3 mgL^−1^) on serum antibacterial activity, antioxidant activity, hematological parameters and growth performance of the crucian carp. The results showed that there were greater antibacterial properties under conditions of hypoxia at 18 °C (L18) and hyperoxia at 24 °C (H24). The activities of catalase, glutathione peroxidase and total superoxide dismutase were the highest at 24 °C under hypoxia and hyperoxia. In addition, the contents of glucose and total protein first increased and then decreased with the change of temperature; triglycerides were the lowest at 30 °C. The blood parameters of the carp were within a normal range at 24 °C; however, the growth rate was at its lowest under hypoxia treatment at 30 °C (L30). This study showed that high temperature impairs the antibacterial ability, antioxidant capacity and growth performance of the crucian carp, and high oxygen levels can alleviate these adverse reactions. This research provides a theoretical basis for subsequent aquaculture studies.

## Introduction

In many European countries, the survival of the crucian carp (carassius) has been affected by the invasion of exotic species such as silver carp and goldfish (Wang et al., 2021a) and it is considered to be a threatened species (Dagoudo et al., 2021). The species grows rapidly in size, however, it suffers from many environmental challenges since its growth is related to temperature and oxygen conditions (Tetreault, Fogle & Guerdat, 2021; Jiang et al., 2021b; Nitz et al., 2020).

Temperature plays a key role in the physiological state and ecosystem function of fish, and maintaining the appropriate temperature is important for aquaculture management (Lindmark, Ohlberger & Gardmark, 2021; Burt, Hinch & Patterson, 2011; O’Gorman et al., 2016; Barbieri, deMedeiros & Henriques, 2016; Barbieri & Bondioli, 2015). Enzyme activity, metabolic rate, and immunoglobulin levels can be affected by sudden changes in temperature, thereby affecting fish survival (Kumar, 2021; Chen et al., 2021; Magouz et al., 2021). Temperature conditions that are not ideal can inhibit growth, decrease antibacterial ability, disrupt the physiological balance, and may even cause death (Wang et al., 2021a; Neves et al., 2021). Oxygen is an indispensable element for every aquatic animal to maintain metabolic activity (Alver, Føre & Alfredsen, 2021), and it is also one of the crucial limiting factors in aquaculture production (Vakili et al., 2021; Lv et al., 2021). The growth parameters, hematological parameters, and apoptotic indices of fish are reduced by hypoxic conditions (Salem, Abdel-Ghany & Almisherfi, 2021; Álvarez et al., 2020; Li et al., 2020). Fish can be subjected to a certain degree of oxidative damage by hypoxia, which impacts growth and serum biochemical indicators, subsequently affecting the metabolism and immune system of the fish (Borowiec & Scott, 2021a).

There is a negative correlation between the temperature of the water and the oxygen content (Jiang et al., 2021b) and some fish may exhibit agitation or avoidance behavior until the water temperature becomes tolerable. The functional activities of the organs and tissues in the fish are altered with the temperature variations so that the fish body can maintain a balanced state as much as possible under higher temperatures (Kochhann et al., 2021). Under hypoxic conditions, fish may move to an environment with sufficient oxygen. In addition, fish can resist hypoxia by adjusting their behavior, such as increasing the oxygen transmission capacity of the operculum (Dawson et al., 2022). The metabolic rate of fish increases with higher temperatures (Zhao et al., 2021), and the growth rate of fish can be reduced by low oxygen conditions (McNicholl et al., 2021). Studies have also shown that hyperoxia conditions can alleviate the heat stress response of fish to a certain extent. The effects of temperature and oxygen alone on the crucian carp have been extensively studied and discussed, however, few experts have studied the effect of the interaction of the two factors on the crucian carp.

The interaction effects of three temperatures (18 °C, 24 °C, and 30 °C) and three oxygen levels (2.1 mgL^−1^, 5.4 mgL^−1^, and 9.3 mgL^−1^) were studied, and the changes in the antibacterial properties, antioxidant capacity, serum biochemistry, blood parameters, and growth parameters of crucian carp were explored under the nine combinations. This research will provide a theoretical basis for an aquaculture environmental control system.

## Materials & Methods

### Fish collection and maintenance

All crucian carp used in this study were provided by Xiong Feng Co., Ltd. (Guangdong, China). The experimental fish were male. Before the experiment, 200 active crucian carp were put into five 400 L circulating water tanks for one week. Temperature controllers were used to keep the water temperature at 17 ± 0.5 °C. The photoperiod was 12:12 h (light/dark), and dissolved oxygen was kept at 6.7 ± 0.1 mgL^−1^. pH was 7.1 ± 0.1, nitrite was 0−0.12 mg/L, and ammonia nitrogen was 0−0.12 mg/L. The water quality parameters were determined by a water quality detection device (Shanghai Lanchang Automation Technology Co., Ltd., Shanghai, China). Fish were fed commercial pellets (protein 35%, fat 5%, ash 15%, and water 12.5%) twice daily (8:30 and 17:30) (Shandong Binzhou Ruixing Biological Technology Co., Ltd., Shandong, China). Fish were fed 5% of the body weight of the shoal. Debris and manure were cleaned and the water was replaced daily with 60% fresh water.

### Trial design

**Figure 1:**
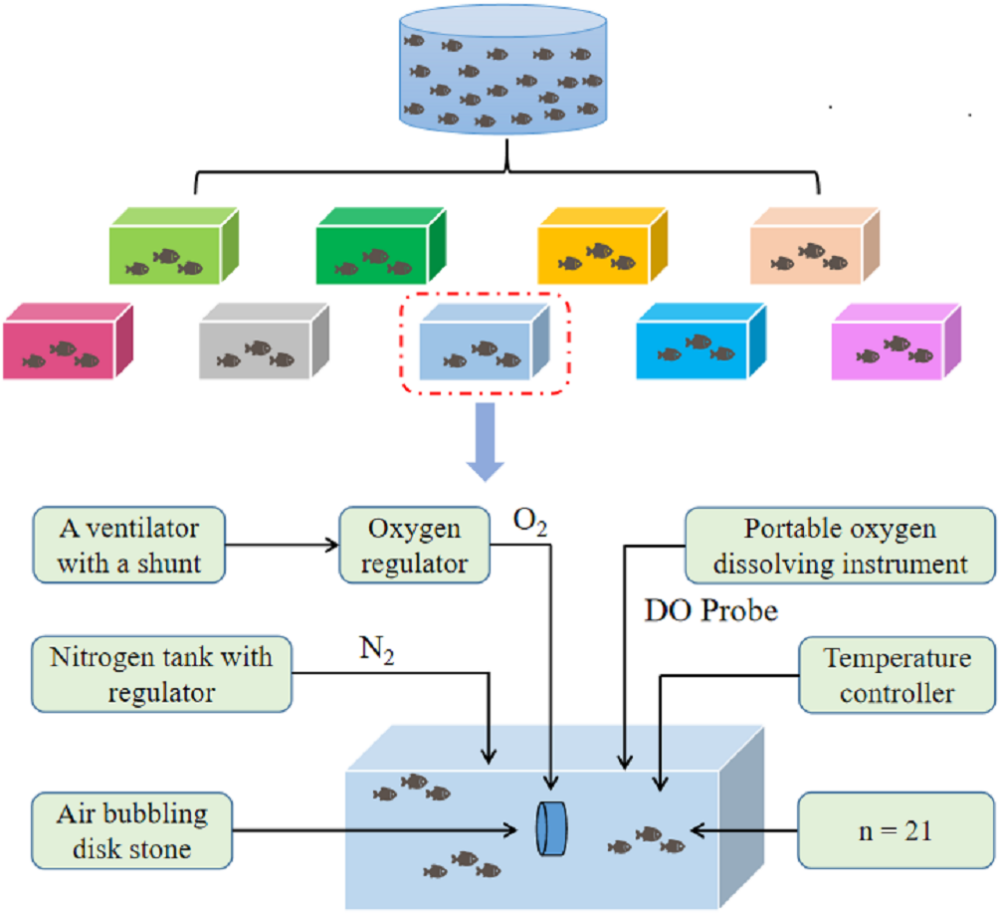
Schematic diagram of oxygen regulation system.

The experimental crucian carp (initial body weight 109.82 ± 2.16 g) chosen from the circulating water tanks were randomly distributed in nine circulating water tanks, with 21 fish in each tank. The size of the tanks were 150 cm ×100 cm ×100 cm. The fish were reared for one week before the experiment. A two-factor, three-level experiment was designed, including temperatures of 18 °C, 24 °C, and 30 °C, and oxygen concentrations of 2.1 mgL^−1^, 5.4 mgL^−1^, and 9.3 mgL^−1^ with three replicates for each treatment. Each tank was divided into three feeding spaces of the same size using two partitions, and there were seven experimental crucian carp in each space. A temperature controller (Guangdong Foshan Lvban Electronic Commerce Co., Ltd., Guangdong, China) was used to gradually change the water temperature from 17 °C to 18 °C, 24 °C, and 30 °C at a rate of 1 °C per hour. In this experiment, hyperoxia conditions were created by increasing the oxygen content, and hypoxic conditions were created by increasing the nitrogen content. Results were analyzed in each trial at 18 °C and the hypoxia group was used as a control group. The schematic diagram of the oxygen regulation system is shown in Fig. 1, and includes an oxygenator, a nitrogen container, a water tank, and a portable dissolved oxygen meter. The trials ran from December 19, 2021 to January 19, 2022, for a total of 30 days.

### Antibacterial experiment

The proliferation ability of bacterial isolates in crucian carp serum was determined using a 96-well plate. Specifically, freshwater bacteria (*Aeromonas*) were utilized to determine the antibacterial capacity in serum samples. *Aeromonas* generally grew normally on agar plates at 25 °C. Fresh single colonies were picked and diluted in five mL of nutrient broth and incubated for 12 h in an orbital incubator at 28 °C with the speed set at 150–200 rpm. Then, the serum antibacterial ability was tested (Dong et al., 2017). Simultaneous serum samples and bacterial suspensions were serially diluted with PBS, twice. Furthermore, 100 µL of bacterial diluent and the same volume of crucian carp serum were added into a 96-well plate. A total of 100 µL of crucian carp serum and 100 µL of nutrient solution were added to the negative wells. A total of 100 µL of bacterial suspension and 100 µL of nutrient broth were added to the positive wells. This experiment was repeated three times. The 96-well plates were incubated at 28 °C. Then, the OD value at 600 nm was measured by a microplate reader with intervals of 1 h, for a total of 12 h measured. The determination of bacterial viability was determined by the ratio between the number of viable bacteria and positive control bacteria, and the results were showed as a percentage (100%).

### Sampling procedures

Food was withheld from the experimental crucian carp for 24 h before sampling, and three experimental fish were randomly selected from each independent area. Each fish was individually sampled and stored. All sampling was performed after MS-222 anesthesia. Blood was drawn from the caudal vessel using a heparinized syringe. One part of the sample was used for the measurement of blood parameters, and the other part was centrifuged at 1, 350 × g for 10 min, and the serum was obtained after coagulation at 4 °C for 5 h. The serum was stored at −20 °C for antibacterial, antioxidant, and biochemical tests.

### Physiological indicators

Three crucian carp were randomly selected from each experimental water tank for physiological and biochemical tests, and the results were expressed as mean ± SD. The data were sorted and analyzed. The total superoxide dismutase (SOD), catalase (CAT), and glutathione peroxidase (GPx) activities were measured with a microplate reader using a SOD assay kit (WST-1 method), a CAT visible light kit, and a GSH-PX assay kit, respectively. The total superoxide dismutase activity was determined by the hydroxylamine method at 550 nm (Li et al., 2013). Catalase activity was measured at 405 nm by the ammonium molybdate method (Adel et al., 2021). Glutathione peroxidase activity was measured colorimetrically at 412 nm (Jiang et al., 2021a). The kit was purchased from Nanjing Jiancheng Bioengineering Institute (Nanjing, China). Blood biochemical parameters (glucose, triglycerides, and total protein) were determined with a Cobas C-311 automatic biochemical analyzer and commercial assay kits. Hematocrit (HCT), mean hemoglobin content (MCH), and hemoglobin (HGB) were detected with a BM830 automatic hematology analyzer.

### Growth performance

(1) \begin{eqnarray*}\text{Specific growth rate (SGR)}=100\text{%}\times ({\text{lnW}}_{2}-{\text{lnW}}_{1}),\end{eqnarray*}

(2) \begin{eqnarray*}\text{Relative growth rate (WGR)}=100\text{%}\times ({\text{W}}_{2}-{\text{W}}_{1})/{\text{W}}_{1},\end{eqnarray*}

(3) \begin{eqnarray*}\text{Survival rate (SR)}=100\text{%}\times ({\text{N}}_{2}/{\text{N}}_{1}),\end{eqnarray*}

W_1_: the initial weight of fish, W_2_: the final weight of fish, N_2_: the final number of fish, N_1_: the initial number of fish.

### Statistical analyses

Data analysis was performed using GraphPad Prism 5.0 software (GraphPad Software Inc., USA). Differences between groups were calculated by multiple comparison test by a two-way ANOVA. *P* < 0.05 indicate a statistical significance. Results were expressed as the mean ± *SD* (*n* = 9).

## Results

### Antibacterial experiment

**Figure 2:**
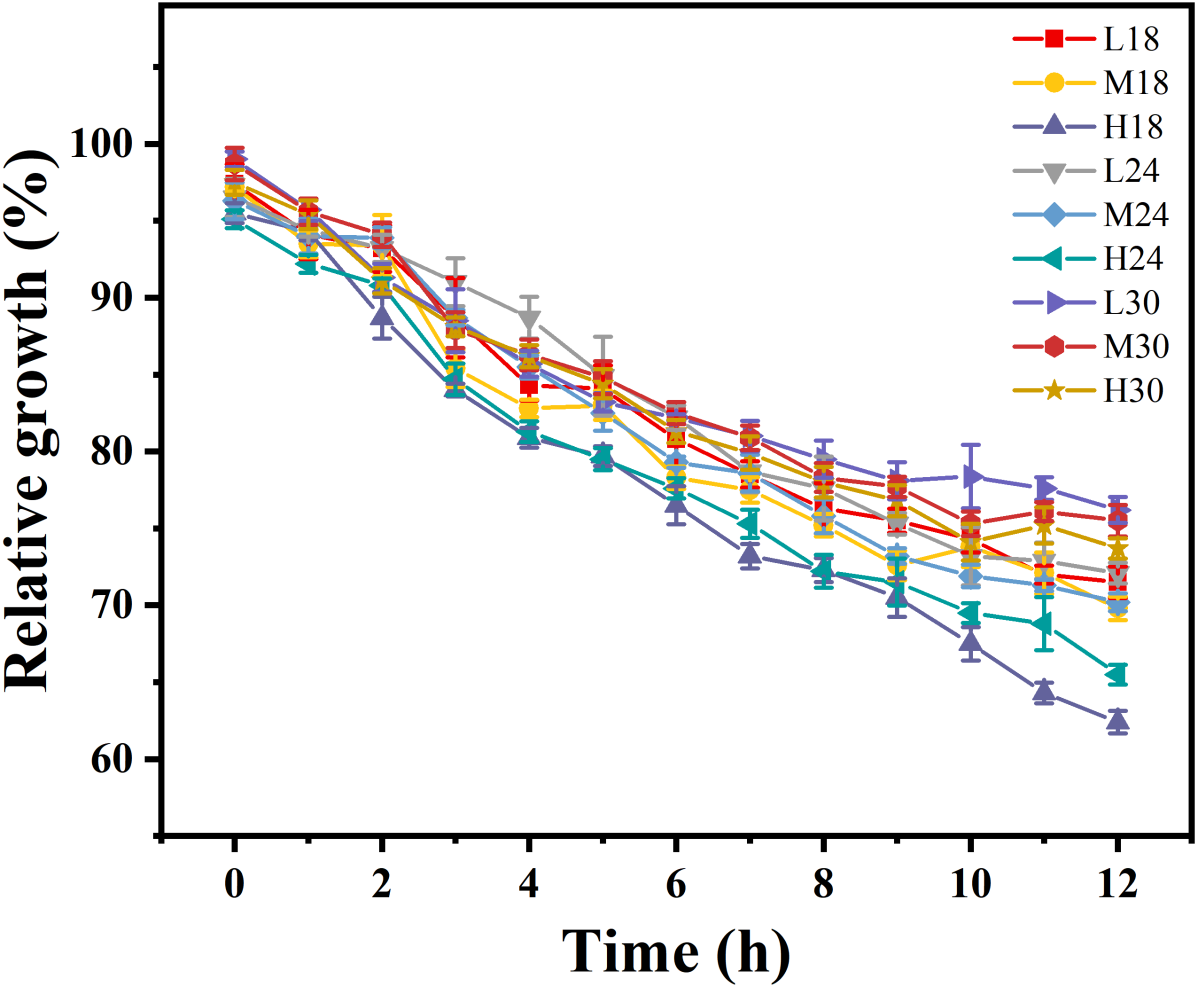
The growth rates of *Aeromonas* in crucian carp serum were compared by two-way ANOVA under the interaction conditions of three temperatures (18 °C, 24 °C, and 30 °C) and three oxygen levels (2.1 mgL ^−1^ , 5.4 mgL ^−1^, and 9.3 mgL ^−1^). Values are expressed as mean ± SD of triplicate pooled data for each treatment. Means of different letters in the same row were significantly different (*P* < 0.05).

We analyzed bacterial growth in the serum sampled from the crucian carp under nine treatments. *Aeromonas* is a general pathogen of aquatic animals. Figure 2 shows a decrease in the relative growth rates of *Aeromonas* in all nine treatment groups over time. Under the same oxygen conditions, the relative growth rate of *Aeromonas* increased with a rise in temperature. The relative growth rate of *Aeromonas* slowed with increased oxygen concentrations. The relative growth rate of the H24 and H18 treatments were low, and the antibacterial effect of serum improved. The relative growth rate of the L30 and L18 treatments were higher, and the serum antibacterial effect was poorer.

### Serum antioxidant test

**Figure 3:**
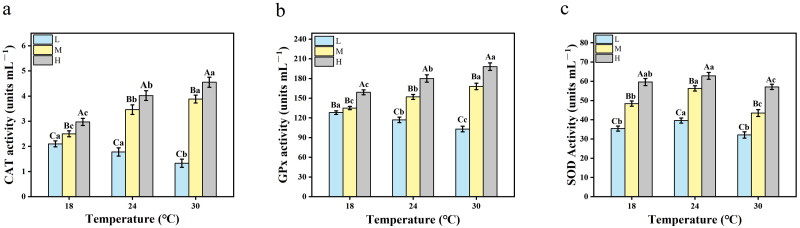
The two-way ANOVA was used to detect the changes of serum antioxidant enzyme activities of crucian carp at different temperatures and oxygen levels (*n* = 9). Capital letters represent significant differences under different oxygen conditions at the same temperature (*P* < 0.05). Lowercase letters represent significant differences under the same oxygen and different temperature conditions (*P* < 0.05). Values are expressed as mean ± SD of triplicate pooled data for each treatment. L represents hypoxic conditions; M represents medium oxygen conditions; H represents hyperoxic conditions; (A) CAT; (B) GPx; and (C) SOD.

To explore the antioxidant capacity of the crucian carp, the activities of three antioxidant enzymes were measured in the serum sampled from nine treatment groups. As shown in Figs. 3A–3C, the activities of CAT, GPx, and SOD in the serum of the crucian carp were significantly enhanced with the increase of oxygen concentration (*P* < 0.0001) under the same temperature conditions. Compared with 18 °C, the serum CAT activity at 24 °C and 30 °C decreased by 15.24% and 36.67% under hypoxic conditions. The serum CAT activity increased by 38.40% and 55.20% at 24 °C and 30 °C when exposed to medium levels of oxygen. When exposed to high oxygen, serum CAT activity increased by 41.41% and 53.20% at 24 °C and 30 °C, as shown in Fig. 3A. Figure 3B shows that under hypoxia, the serum GPx activity of the crucian carp under 24 °C and 30 °C decreased by 4.76% and 21.43%, respectively, compared with 18 °C. Under medium oxygen conditions, the serum GPx activity of the crucian carp at 24 °C and 30 °C increased by 11.85% and 24.44%, respectively, compared with 18 °C. Under high oxygen conditions, the serum GPx activity at 24 °C and 30 °C increased by 16.46% and 25.32%, respectively, compared with 18 °C. Figure 3C shows that under hypoxic condition, the serum SOD activity of the crucian carp increased by 11.86% and decreased by 9.32% at 24 °C and 30 °C, compared with 18 °C. Under medium oxygen conditions, the serum SOD activity increased by 16.32% and 10.10% at 24 °C and 30 °C, compared with 18 °C. Under high oxygen conditions, the SOD activity of the serum increased by 5.37% and decreased by 4.19% at 24 °C and 30 °C, compared with 18 °C.

### Serum biochemical experiment

**Figure 4:**
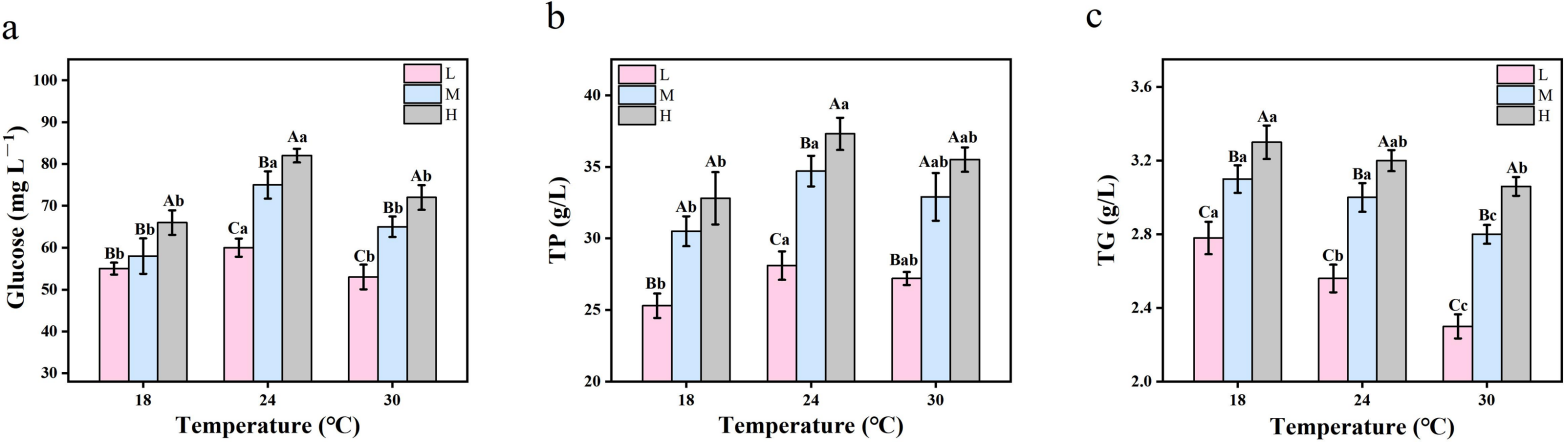
The two-way ANOVA was used to detect the changes of serum biochemical parameters of crucian carp at different temperatures and oxygen levels (*n* = 9). Capital letters represent significant differences under different oxygen conditions at the same temperature (*P* < 0.05). Lowercase letters represent significant differences under the same oxygen and different temperature conditions (*P* < 0.05). Values are expressed as mean ± SD of triplicate pooled data for each treatment. L represents hypoxic conditions; M represents medium oxygen conditions; H represents hyperoxic conditions. TP, total protein, TG, triglycerides. (A) glucose; (B) TP; and (C) TG.

Figure 4A shows that with the increase of oxygen concentration, glucose levels increased significantly under the three temperature conditions. At the same oxygen concentration, the glucose level of the crucian carp increased and then decreased with the increase of temperature, reaching the highest levels at 24 °C (*P* < 0.05). Figure 4B shows the change of total protein content in crucian carp serum. Under the same oxygen concentrations, the total protein content at 24 °C was higher than that at 18 °C and 30 °C. The total protein content under hypoxic conditions was lower than that under medium oxygen and hyperoxia conditions (*P* < 0.0001). Changes in the serum triglyceride content of crucian carp are shown in Fig. 4C. At the same temperature, with the increase of oxygen concentration, hyperoxia produced greater triglyceride contents than medium oxygen and hypoxic conditions. When oxygen concentrations were the same, the triglyceride content decreased with increasing temperatures (*P* < 0.05).

### Hematological analysis

**Figure 5:**
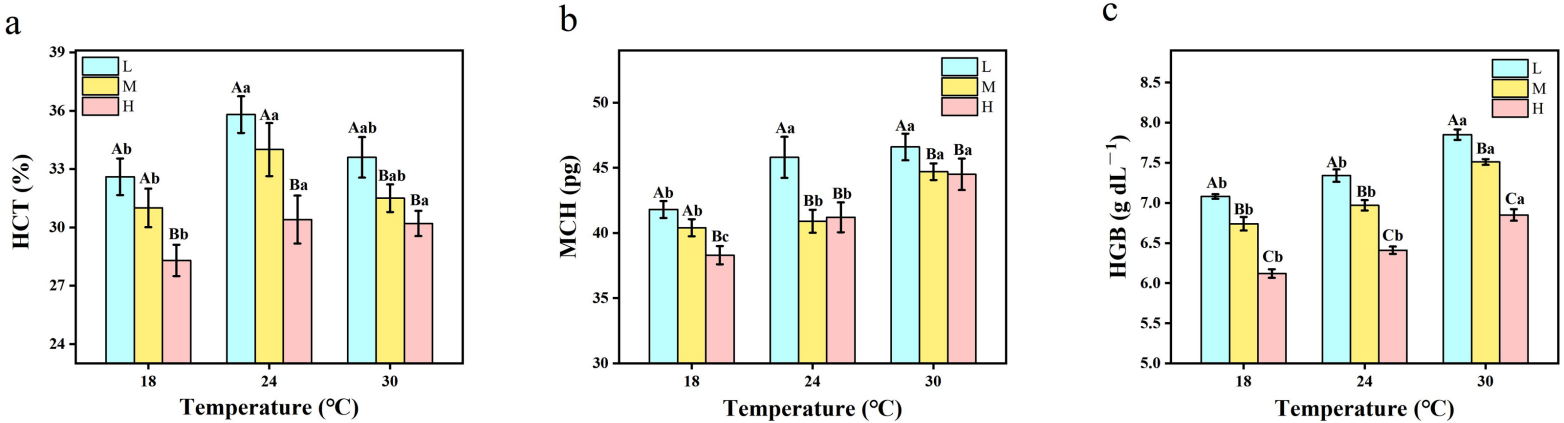
The two-way ANOVA was used to detect the changes of hematological variables in crucian carp at different temperatures and oxygen levels (*n* = 9). Different capital letters indicate significant differences (*P* < 0.05) under different oxygen conditions at the same temperature. Different lowercase letters indicate significant differences in the same oxygen at different temperatures (*P* < 0.05). Values are expressed as mean ± SD of triplicate pooled data for each treatment. L represents hypoxic conditions; M represents medium oxygen conditions; H represents hyperoxic conditions. HCT, hematocrit; MCH, mean hemoglobin content; HGB, hemoglobin. (A) HCT; (B) MCH; and (C) HGB.

The differences in blood HCT, MCH and HGB levels are shown in Fig. 5. As shown in Fig. 5A, HCT at 24 °C was significantly higher than that at 18 °C and 30 °C. At the same temperature, the HCT of hypoxia was higher than that of hyperoxia and moderate oxygen (*P* < 0.01). Figure 5B shows that under the same oxygen concentration, MCH at 30 °C was significantly higher than that at 18 °C, and slightly higher than at 24 °C. Under the same temperature conditions, MCH under hypoxic conditions was significantly higher than that under hyperoxia and medium oxygen condition (*P* < 0.05). As shown in Fig. 5C, at the same temperature, HGB under hypoxic conditions was higher than that under medium oxygen and hyperoxia conditions. At the same oxygen concentration, HGB at 18 °C was slightly lower than that at 24 °C and 30 °C (*P* < 0.0001).

### Growth performance

The FW, SGR, WGR, and SR of crucian carp after different treatments are shown in Table 1. The FW of crucian carp was different between different treatments of temperature and oxygen concentrations. Under hypoxic conditions, the FW of crucian carp in L30 was lower than that in L18 and L24 (*P* < 0.01). Under medium oxygen conditions, the FW of the M30 crucian carp was lower than that of M18 and M24. Under hyperoxia conditions, the FW of the H30 crucian carp was significantly lower than that of the H18 and H24 treatments (*P* < 0.05). In addition, under the same temperature conditions, the FW of crucian carp under hyperoxia was significantly higher than that under hypoxia. According to the analysis results, H24 (136.71 ± 2.51 g) and L30 (105.86 ± 2.96 g) were the highest and lowest values of the FW. When the temperature remained the same, the SGR of the crucian carp under hyperoxia was significantly higher than that under mesoxia and hypoxia (*P* < 0.01). At static oxygen levels, the SGR of the crucian carp reached the highest at 24 °C and the lowest at 21 °C (*P* < 0.01). The SGR of the crucian carp in the H24 and M24 treatments were the highest, at 0.68% and 0.63%, respectively. When the oxygen concentration was same, the WGR of crucian carp under 18 °C was lower than that at 24 °C and 30 °C. When the temperature was same, the WGR of crucian carp under hyperoxia was significantly higher than that under medium oxygen and hypoxia (*P* < 0.0001). The carp in H24 and M24 had the highest WGR. The SR of crucian carp was affected by temperature. When the oxygen concentration was same, the SR was relatively high at 18 °C and 24 °C, and the SR was relatively low at 30 °C. The SR was most affected by oxygen concentration, and the SR of H24 and H18 was 100%, respectively. The SR under L30 was the lowest, at 71.43%.

**Table 1:** Summary of the two-way ANOVA results for the growth performance of crucian carp under different interaction treatments. Values are expressed as mean ± SD of triplicate pooled data for each treatment. Means in the same column with different letters are significantly different (*P* < 0.05).

**Treatment**	**IW (g)**	**FW (g)**	**SGR (%)**	**WGR (%)**	**SR (%)**
L18	112.05 ± 2.13	116.10 ± 2.22Bb	0.12 ± 0.04Cb	3.62 ± 1.13Cb	80.95 ± 6.73
M18	112.43 ± 2.48	124.10 ± 2.81Bb	0.33 ± 0.08Bb	10.39 ± 2.69Bb	95.24 ± 6.73
H18	112.86 ± 2.23	134.60 ± 3.10Ab	0.59 ± 0.08Ab	19.25 ± 2.95Ab	100.00 ± 0.00
L24	109.20 ± 3.10	125.05 ± 3.20Ca	0.45 ± 0.13Ca	14.61 ± 4.58Ca	76.19 ± 6.73
M24	110.62 ± 2.72	133.52 ± 3.81Ba	0.63 ± 0.10Ba	20.74 ± 3.76Ba	95.24 ± 6.73
H24	111.48 ± 2.97	136.71 ± 2.51Aa	0.68 ± 0.13Aa	22.72 ± 4.71Aa	100.00 ± 0.00
L30	104.20 ± 2.91	105.86 ± 2.96Cc	0.05 ± 0.02Cc	1.59 ± 0.77Cc	71.43 ± 0.00
M30	105.43 ± 2.84	114.40 ± 2.75Bc	0.27 ± 0.07Bc	8.52 ± 2.34Bc	90.48 ± 6.73
H30	109.90 ± 3.01	127.38 ± 2.44Ac	0.49 ± 0.09Ac	15.90 ± 3.43Ac	95.24 ± 6.73

## Discussion

Fish serum is considered to be one of the most commonly used methods to assess the physiological state and health status of fish (Witeska et al., 2022). It contains a variety of immune molecules, such as immune proteins, lysozyme, etc., with high antibacterial properties (Ahmed et al., 2021). In the range of 18−30 °C, when the oxygen concentration was the same, the enzyme activity gradually increased with the increase of temperature. The antibacterial activity of the crucian carp serum also gradually increased. However, the bacteriostatic effect of low temperature was better than that of moderate and high temperatures, because the optimal growth temperature of *Aeromonas* is 22 °C–28 °C (Grilo et al., 2021). At a static temperature, an increased oxygen concentration caused the relative growth rate of *Aeromonas* to decrease. When the amount of oxygen increased, the respiration and metabolic rate of the crucian carp increased and the immune system of the fish worked more efficiently, so that the antibacterial ability was enhanced. Therefore, H18 and H24 were found to have the strongest antibacterial properties.

The growth performance and metabolism of fish are affected not only by temperature and oxygen, but also by their antioxidant defense system (Ritchie & Friesen, 2022). When the temperature and oxygen content change, excessive oxygen free radicals are generated in the environment, and macromolecules such as proteins and lipids in the fish are destroyed, thereby affecting their health (Liu et al., 2021; Aswathy et al., 2021; Baldissera et al., 2020). However, this oxygen radical can be eliminated by the antioxidant defense system of fish, including CAT, GPx, and SOD (Baldissera et al., 2019). Under anoxic conditions, the activities of CAT and GPx in the crucian carp were slightly weakened at 18 °C–30 °C, the SOD activity was slightly increased at 24 °C, and decreased slightly at 30 °C. This means that the crucian carp was in relatively good condition. The activities of these two enzymes were significantly enhanced under mesoxia and hyperoxia conditions. At the same temperature, the activities of the three antioxidant enzymes were the highest in hyperoxia, followed by mesoxia, and lowest in hypoxia. This indicates that the antioxidant system of crucian carp can be activated by conditions of hyperoxia and mesoxia. However, the enzyme activity is extremely low under hypoxic conditions, and the antioxidant capacity is the weakest (Yang et al., 2015). The heat stress experienced by crucian carp may also improve under hypoxic conditions.

Glucose is the main substance for cell and tissue metabolism and physiological activities. Glucose levels in fish are affected by temperature and oxygen levels (da Costa Barroso, Almeida-Val & Luis Val, 2020). Low glucose concentrations can affect the growth rate and appetite of fish (Zhang et al., 2022). The results revealed that the L27-treated crucian carp had the lowest glucose concentrations, which resulted in decreased appetite and slower growth. The main function of serum proteins is to repair damaged tissues and participate in the regulation of plasma osmotic pressure (Siddik et al., 2022). Under the same oxygen concentrations, the total protein activity increased at 24 °C and 30 °C, indicating that the body tissue of crucian carp may have been damaged when the water temperature increased (Wang et al., 2021b). In addition, the total protein content was lower under hypoxic conditions than under meso- and hyperoxia conditions. Therefore, high concentration of oxygen may reduce the degree of tissue damage and accelerate tissue recovery in crucian carp. Oxidized triglycerides are an energy source for fish metabolism (Moorhead et al., 2021). Compared with 18 °C and 24 °C, the survival rate of crucian carp at 30 °C was the lowest, indicating that crucian carp needs to consume a lot of triglycerides under high temperature conditions. At the same temperature, there was less of a reduction of triglycerides under hyperoxia conditions than that under mesoxia and hypoxia conditions. This suggests that hyperoxia helps to alleviate the high temperature-induced triglyceride consumption in crucian carp.

Studies have shown that hematocrit is one of the important blood parameters that reflect the health of fish. At the same temperature, the hematocrit under hypoxic conditions was higher than that under mesoxia and hyperoxia conditions (Gomez Isaza, Cramp & Franklin, 2021). Low oxygen concentrations cause the hemoglobin and oxygen transport capacity in the body to increase and the body is able to maintain normal physiological functions (Borowiec & Scott, 2021b). When the oxygen concentration is too high, although the oxygen carrying capacity is strong, the blood viscosity increases, the blood flow resistance increases, and the hematocrit decreases. Under the condition of constant oxygen conditions, the body temperature increases and the metabolic capacity increases. At this time, the content of hemoglobin increases, the hematocrit increases, and the physiological function of the body can be regulated to a certain extent through this cycle (Schwieterman et al., 2021). Conversely, when the body temperature is low, the utilization of oxygen may be affected, the synthesis of hemoglobin is reduced, and the hematocrit is also reduced. The experimental results are consistent with this conclusion.

The overall health of fish is directly reflected in their growth performance and survival rate. Our test results are shown in Table 1. The survival rate of crucian carp under hyperoxia conditions was significantly higher than that under mesoxia and hypoxia conditions. The growth indexes SGR and WGR were the highest at 24 °C, followed by 30 °C, and the lowest at 18 °C for the crucian carp. The optimal growth temperature was 25 °C, which is similar to the results of previous studies (Sikorska et al., 2018). When the ambient temperature was the same, the growth under hyperoxia conditions was higher than that under mesoxia and hypoxia conditions. Therefore, growth may be inhibited by hypoxia (Beemelmanns et al., 2021; Phu et al., 2022). It was also reported that the growth rate of the crucian carp showed a trend of increasing first and then decreasing within a specific range (Fey & Greszkiewicz, 2021). The results showed that the growth rate of the H24 crucian carp was the highest, and the growth rate of the L30 carp was the lowest. In addition, when the temperature was set to 30 °C, the growth rate under hyperoxia conditions was higher than that of mesoxia and hypoxic conditions, suggesting that hyperoxia may protect the crucian carp from the adverse effects of high temperature.

## Conclusions

Crucian carp had better growth performance under hyperoxia (9.3 mgL^−1^) than under medium oxygen (5.4 mgL^−1^) or hypoxic (2.1 mgL^−1^) conditions. High temperature and hypoxia stress may reduce the antibacterial properties of crucian carp, and various physiological indicators may become disordered as a result. However, these adverse effects can be mitigated to some extent by increasing the oxygen concentration. Therefore, according to the changing trends of various parameters in the nine treatment groups, the optimal growth environment for crucian carp was the H24 treatment. These results indicate that under high oxygen conditions, the water temperature was best kept at 24 °C.

This study was limited by a small sample size, which may have affected the accuracy of the conclusions. Therefore, future studies with a larger sample size are required for further verification.

##  Supplemental Information

10.7717/peerj.14530/supp-1 Supplemental Information 1 The original data of serum antibacterial activity, serum antioxidant activity, serum biochemical, hematological parameters, initial body weight, final body weight and mortality of crucian carp

10.7717/peerj.14530/supp-2 Supplemental Information 2 Author Checklist

## Additional Information and Declarations

### Data Availability

The following information was supplied regarding data availability: The raw data is available in the Supplemental File.
